# Still’s Disease and Autoinflammation: Positioning an Inflammatory Syndrome on the Autoinflammation-Autoimmunity Spectrum

**DOI:** 10.1007/s11926-026-01210-6

**Published:** 2026-02-02

**Authors:** Daniel Pietsch, Sinisa Savic

**Affiliations:** 1 Department of Clinical Immunology and Allergy, St. James’s University Hospital, Leeds, UK; 2 Division of Rheumatology and Clinical Immunology, Medical University of Graz, Graz, Austria; 3 Leeds Institute of Rheumatic and Musculoskeletal Medicine, University of Leeds, Leeds, UK; 4 NIHR Leeds Biomedical Research Centre, Leeds, UK

**Keywords:** Still’s disease, Adult-onset still’s disease, Systemic juvenile idiopathic arthritis, Autoinflammation, Autoimmunity, Macrophage activation syndrome, Cytokine- targeted therapy

## Abstract

**Purpose of this Review:**

Still’s disease exemplifies systemic inflammatory disorders existing on a continuum between autoinflammation and autoimmunity. This review examines Still’s disease through this spectrum lens, integrating recent advances in pathogenesis, clinical heterogeneity, and therapeutic approaches.

**Recent Findings:**

Emerging mechanistic insights reveal complex innate-adaptive immune interactions. Type I interferon signalling and neutrophil extracellular trap formation drive inflammation, while hyperferritinemia actively perpetuates disease through Msr1-mediated signaling. mTORC1 has emerged as a central integration hub converging multiple cytokine signals. Adaptive mechanisms increasingly contribute to complications: both macrophage activation syndrome and lung disease demonstrate IFNγ-dominant pathology with T cell hyperactivation. Clinical phenotyping identifies distinct patient clusters—from hyperferritinemic monocyclic to catastrophic multiorgan phenotypes—reflecting varying innate-adaptive contributions. Current classification criteria permit considerable diagnostic latitude and may inadvertently group mechanistically distinct conditions under a single diagnostic label. IL-1 and IL-6 receptor blockade remain therapeutic cornerstones, with evidence supporting early intervention during a window of opportunity. Novel approaches including IL-18 binding protein, JAK inhibitors, and IFNγ blockade show promise in refractory disease.

**Summary:**

Still's disease predominantly reflects autoinflammatory pathology driven by innate immune dysregulation, yet adaptive mechanisms contribute meaningfully to disease heterogeneity and complications. Recognition as a spectrum disorder—with variable innate-adaptive contributions across patients and disease phases—supports unification of pediatric and adult forms, guides mechanistically targeted therapies, and emphasizes the need for biomarker-driven patient stratification to enable personalized treatment approaches.

## Introduction

Systemic inflammatory disorders are increasingly recognized to exist along a continuum between autoimmunity and autoinflammation, rather than as distinct categorical entities. At one end of this spectrum lie rare monogenetic autoimmune diseases characterized by adaptive immune dysregulation and autoantibody production, while at the opposite end are monogenetic autoinflammatory disorders driven primarily by innate immune activation. Most of systemic inflammatory disorders occupy intermediate positions along this spectrum, exhibiting features of both innate and adaptive immune dysregulation [1].

Still’s disease (SD) is predominantly classified toward the autoinflammatory end of this spectrum, characterized by systemic inflammation, neutrophilic activation, and elevation of innate immune mediators such as IL-1β and IL-18 [2]. However, the presence of adaptive immune features—including T cell activation in complications such as Macrophage activation syndrome (MAS), suggests a more nuanced position within this framework [3].

As a syndromic diagnosis, SD likely represents an umbrella term encompassing multiple distinct pathogenic processes that converge on similar clinical presentations. Current classification criteria, while useful for clinical diagnosis, may inadvertently group heterogeneous disease entities under a single diagnostic label, potentially obscuring important pathomechanistic distinctions along the autoimmune-autoinflammatory spectrum.

This review explores Still’s disease through the lens of autoinflammation, examining where mechanisms of innate immunity dominate and where adaptive features emerge, while incorporating recent insights into disease pathogenesis, clinical heterogeneity and organ manifestations. Throughout, we use the term Still’s disease to encompass both systemic juvenile idiopathic arthritis (sJIA) and adult-onset Still’s disease (AOSD), recently reunified under EULAR 2024 recommendations, reflecting their presumed shared pathophysiology despite clinical heterogeneity [4].

## Genetics : Bridging Innate and Adaptive Susceptibility

In contrast to monogenetic autoinflammatory diseases, such as Familial Mediterranean Fever, Still’s disease does not have a pathognomonic genetic defect. It is therefore considered a complex autoinflammatory disease, in which an interplay between genetic susceptibility and environmental factors lead to emergence of the disease process.

### The Adaptive Side

Interestingly, strong associations were found in genes that encode for proteins we consider part of the adaptive immune system, specifically MHC class 1 & 2 molecules, that are being utilised by antigen- presenting cells to present peptide antigens to T Cells. The HLA class II region consistently shows the strongest genetic association with Still’s disease across populations, with the primary risk localized to different genes: DRB1*11 in multi-ethnic pediatric cohorts, the DRA-DRB5 intergenic region in individuals of Chinese ancestry, and DRB1*15:01 in those of Japanese ancestry [5–7]. Notably, HLA-DRB1*15 has emerged as a key risk factor for severe complications, being strongly associated with parenchymal lung disease, drug hypersensitivity reactions to IL-1/IL-6 inhibitors, and pulmonary arterial hypertension [8, 9].

Such associations are characteristic of autoimmune diseases, suggesting involvement of T cell-mediated adaptive immunity despite the predominantly autoinflammatory clinical phenotype and absence of typical pathogenic autoantibodies in most cases.

Beyond classical HLA alleles, a functional polymorphism in LILRA3, encoding an HLA class I receptor, confers increased SD susceptibility in adults and is associated with neutrophilia and enhanced neutrophil extracellular trap formation, linking HLA-related genetic to innate immune dysregulation [10].

### The Innate Side

On the innate immune side, a few studies have found variants in genes responsible for monogenetic autoinflammatory diseases, such as *MEFV*, *NLRP3 or TNFRSF1A*. While such variants are found in a substantial minority of SD patients (15–20% in some cohorts), only a subset are classified as pathogenic or likely pathogenic, and most show incomplete penetrance [11–15]. Additionally, functional polymorphisms in macrophage migration inhibitory factor (MIF), an upstream regulator of pro-inflammatory cytokines, have been associated with adult SD susceptibility and plasma MIF levels [16].

More recently, a study found enrichment of rare HLH variants in paediatric SD patients, even in those who didn’t develop secondary HLH/MAS. However, none of these variants were classified as pathogenic, which raises questions about their functional significance and whether they contribute to disease through mechanisms distinct from classical HLH pathophysiology [17].

In addition, in paediatric Still’s disease, polymorphisms in the IL10 gene family are associated with disease susceptibility, suggesting impaired anti-inflammatory responses [18].

### The Acquired Side

More recently, the contribution of clonal haematopoesis in autoinflammatory diseases has come to attention due to the discovery of diseases such as VEXAS syndrome, which is a disease that is driven by myeloid cell clones that arise due to somatic mutations in the UBA1 gene [19]. It is also well established that in clonal haematopoiesis of indeterminate potential (CHIP), a pre-malignant state characterised by clonal expansion of hematopoietic stem and progenitor cells harboring somatic mutations in epigenetic regulators and other driver genes, the affected myeloid cells can acquire a more inflammatory phenotype, in part due to enhanced inflammasome activation and increased production of pro-inflammatory cytokines [20]. In adult SD, patients have shown an earlier onset of somatic variants in genes that are associated with CHIP compared to healthy controls [13, 21]. It is unlikely that this presents a massive contributing factor in paediatric SD, as these mutations typically accumulate during adulthood, although the above mentioned study has shown putative somatic variants in CHIP genes in patients of the age cohort 19–29 years [13]. Nevertheless, the unspecific activation of innate immune system via these acquired mutations is likely one of the multiple factors contributing to the development of the disease in a proportion of patients.

**Fig. 1:**
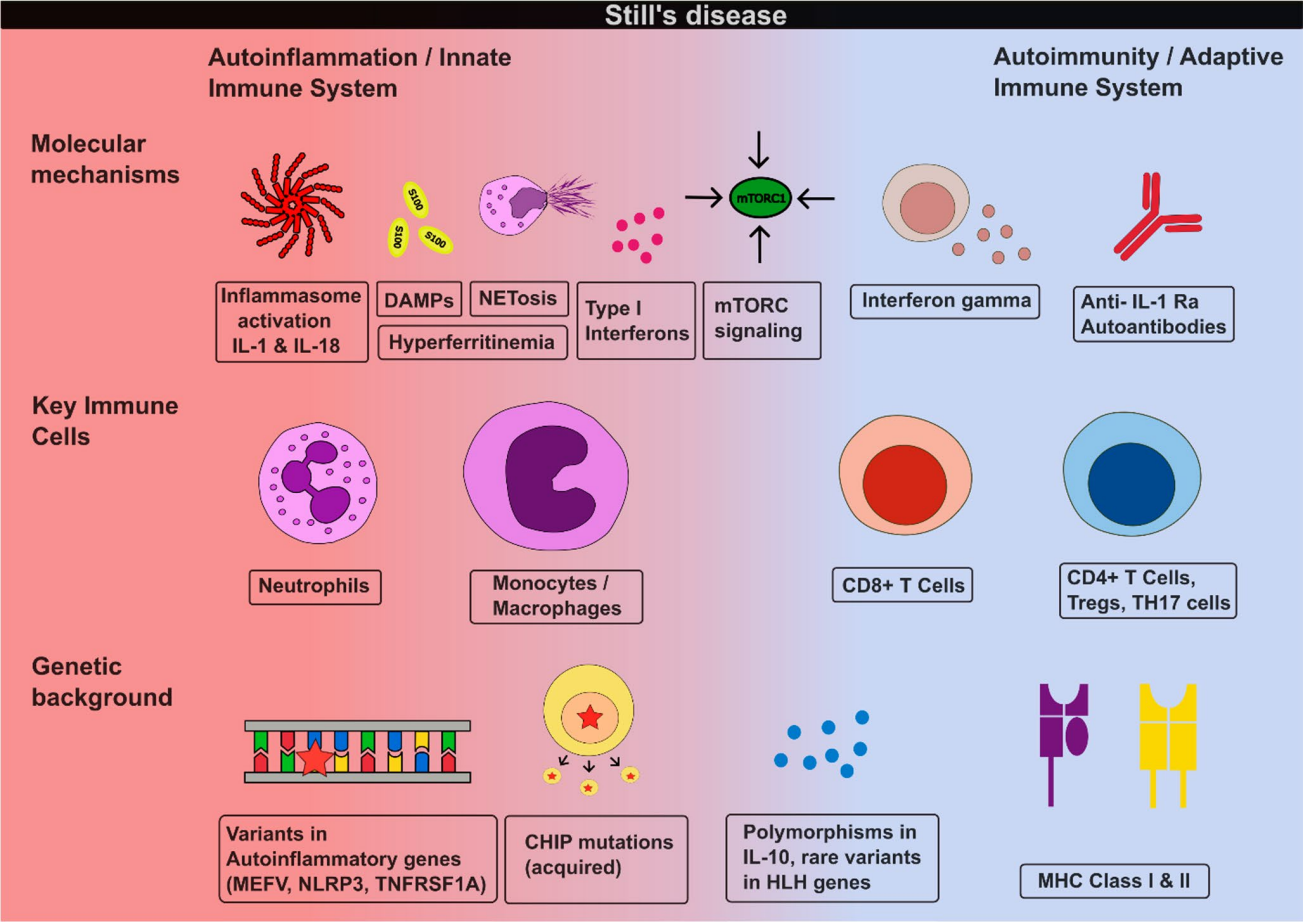
Still’s disease across the autoinflammatory-autoimmune spectrum. The figure illustrates genetic susceptibility factors, key cellular mediators, and molecular mechanisms contributing to disease pathogenesis. From bottom to top: genetic factors range from autoinflammatory gene variants (left) to MHC associations (right); cellular mediators progress from innate (neutrophils, macrophages) to adaptive (T cell subsets); molecular mechanisms include both innate pathways (inflammasome, NETs, DAMPs) and adaptive features (interferons, autoantibodies). mTORC1 serves as a central integration hub

The absence of a unifying genetic defect, combined with diverse associations spanning HLA alleles, inflammasome genes, and acquired somatic mutations, reinforces the concept of Still’s disease as a syndromic diagnosis encompassing multiple pathogenic pathways (Table 1; Fig. 1).

**Table 1:** Genetic associations in still’s disease across the immune spectrum

Immune Component	Gene(s)	Representative Studies	Key Findings
Innate Immune System			
Inflammasome	MEFV, NLRP3	Nonaka et al. 2015 (*n* = 49, Japanese); Asano 2017 (*n* = 87, Japanese); Sighart et al. 2018 (*n* = 40, German) García-Melchor 2014 (*n* = 18, Spanish)	**MEFV - Exon 10 variants (M694I**,** G632S)** enriched in Japanese AOSD (6.1% vs. 0%, *P* = 0.031, Nonaka 2015). MEFV variants found in 56.3% Japanese AOSD patients, modulate HLA-DR5 risk (Asano 2017). Rare likely pathogenic MEFV variants in 7.5% German AOSD (3/40, pc = 2.34E − 03), 2/3 carriers received biologics (Sighart 2018). **NLRP3**: VUS in 1/40 (Sighart 2018). 1 low-penetrance variant and 1 polymorphism (Garcia-Melchor 2014)
Pattern recognition, TNF signalling, Other	TNFRSF1A, NOD2, TNFAIP3, MVK, SCN9A	Sighart et al. 2018 (*n* = 40, German), Prieto-Peña et al. 2024 (*n* = 24, Spanish), García-Melchor 2014 (*n* = 18, Spanish)	**TNFRSF1A**: 2/40, 1 likely pathogenic and 1 pathogenic variant (Sighart 2018). 1 VUS (Prieto-Peña 2024). **TNFAIP3 & SCN9A**: 1 VUS (Prieto-Peña 2024) Carriers of VUS showed atypical manifestations and therapeutic refractoriness (Prieto-Peña 2024). **NOD2**: 2/24 VUS (Prieto-Peña 2024). p.R702W polymorphism (2/18 patients) (García-Melchor 2014).
Anti-inflammatory Regulation	IL-10, IL-10RA	Fife et al. 2006 (*n* = 172, UK)	Low IL10-producing allele (IL10-1082 A) associated with sJIA (*p* = 0.031).
Upstream Cytokine Regulation	MIF	Wang et al. 2013 (*n* = 100, Chinese)	MIF − 794 CATT5 allele and − 173*C/−794 CATT5 haplotype associated with AOSD susceptibility (OR 5.53, *p* = 0.001) and elevated plasma MIF levels.
Cytotoxic Function			
Granule exocytosis	STXBP2, UNC13D, LYST, PRF1	Correia Marques et al. 2024 (*n* = 480 sJIA, INCHARGE)	Rare **STXBP2** and **UNC13D** variants enriched in sJIA. UNC13D specifically associated with MAS subgroup (*P* = 0.0047). Digenic HLH combinations more common in sJIA (2.7% vs. 1.1%, *P* = 0.007).
Complex/Mixed Mechanisms			
Somatic + Germline	CHIP genes (DNMT3A, TET2, ASXL1); Autoinflammation genes (ALPK1, PLCG2, TRAP1, NOD2)	Topping et al. 2025 (*n* = 60, European WES)	51.7% carried multiple rare germline variants (across autoinflammation, CHIP, and type I interferonopathy genes), 30.0% putative somatic variants, 23.3% both. Autoinflammatory gene variants: 33.3% (cohort #2) and 43.4% (cohort #3) vs. 20.4% controls.
Adaptive Immune System			
MHC Class I & II	HLA-DRB1*11, *15, *09, *12, *04; HLA-DRA/DRB5; HLA-G; LILRA3	Ombrello et al. 2015 (*n* = 770 sJIA); Li et al. 2020 (*n* = 264, Chinese GWAS); Asano et al. 2017 (*n* = 96, Japanese); Boucly et al. 2025 (*n* = 11 PAH); Wang et al. (*n* = 164, Chinese)	HLA-DRB1*11: Associated with sJIA across 9 populations (OR 2.3, *P* = 2.7 × 10⁻¹⁶, Ombrello 2015). **HLA-DRA/DRB5-** intergenic region: strongest genome-wide significant association in the cohort (OR 2.36, *p* = 4.10 × 10⁻¹⁹; Li 2020). HLA-DRB1*15:01: Linked to MAS and severe disease (OR 3.04, Asano 2017); enriched in SD patients with PAH vs. SD patients without PAH (72.7% vs. 30.1%, *P* = 0.014, Boucly 2025). **HLA-G**: Genome-wide significance (OR 2.14, *P* = 1.97 × 10⁻⁸, Li 2020). HLA-DRB1*09:01: Protective in Japanese cohort (OR 0.34, Asano 2017). **LILRA3**: Functional LILRA3 associated with AOSD (OR 2.09, *p* = 0.034), neutrophilia, and enhanced NET formation (Wang 2021).

Studies are ordered by immune component. Key findings include effect sizes where reported. Odds ratios (ORs) vs. healthy controls unless otherwise stated. AOSD, adult-onset Still’s disease; CHIP, clonal hematopoiesis of indeterminate potential; GWAS, genome-wide association study; HLH, hemophagocytic lymphohistiocytosis; MAS, macrophage activation syndrome; MHC, major histocompatibility complex; NS, not significant; PAH, pulmonary arterial hypertension; sJIA, systemic juvenile idiopathic arthritis; VUS, variant of uncertain significance; WES, whole exome sequencing

## Pathomechanisms Along the Spectrum

### Initiation of SD

A frequently discussed working hypothesis is that of “second hits” acting on a background of genetic susceptibility, a concept that is relevant to many complex autoinflammatory and autoimmune diseases [22]. In this model, an individual with a predisposing genetic background experiences a disruption of immune homeostasis that initiates an aberrant inflammatory response. In SD, potential triggers include viral or bacterial infections [23], while in some cases the disease process was driven by an underlying malignancy [24]. Some investigators further propose that the disease process is initially fueled by activation of the innate immune system and subsequently amplified by adaptive mechanisms—a sequence that might be interrupted in its early phases during the so-called “window of opportunity” [25, 26].

### Autoinflammatory Core: Innate Immune Activation

As for all diseases on the autoinflammatory side of the spectrum, innate immune cells, such as neutrophils and monocytes/macrophages play a prominent role in the initiation as well as maintenance of the disease. Below described disease mechanisms, such as inflammasome activation or neutrophil extracellular traps, are predominantly characteristics of these cell types.

Neutrophils and monocytes/macrophages are key innate immune cells in SD, and their role and activation mechanisms have been reviewed extensively elsewhere [27]. We will therefore restrict ourselves to the already known, as well as the more recently described mechanisms in the pathogenesis of SD.

### Inflammasome Activation

Inflammasome activation has been shown to play an important role in SD, as evidenced by dysregulated IL-1 production in paediatric Still’s disease, which provided the underlying rationale for the use of IL-1 inhibitors such as Anakinra, which is also one of the mainstays of the current treatment guidelines [4, 28]. Furthermore, serum levels of Gasdermin D, a pore forming protein which gets activated via inflammasome activation and facilitates release of IL-1 and IL-18, as well as pyroptosis, were elevated in SD patients compared to healthy individuals. In a mouse model, the genetic deletion or inhibition of Gasdermin D could improve clinical symptoms of TLR9- induced macrophage activation syndrome [29]. There have been studies looking at the clinical utility of the readouts of inflammasome activation, that showed significantly increased IL-18 levels in the serum of SD patients, similar to that of monogenetic AIDs like FMF, that have a genetically determined overactivation of the inflammasome [2].

### Damage-Associated Molecular Patterns and Alarmin Release

Beyond canonical cytokines, Still’s disease is characterized by excessive release of damage-associated molecular patterns (DAMPS). The S100 protein family serve as endogenous alarmins secreted by activated neutrophils and monocytes that correlate strongly with disease activity, ferritin levels, and proinflammatory cytokines [30, 31]. These proteins actively perpetuate inflammation through TLR4-mediated signalling and MAPK pathway activation, creating self-sustaining inflammatory loops [30, 32]. Automated S100A8/A9 assays can distinguish Still’s disease from infections with high specificity [33].

### Type I Interferon & NET Formation

Recent studies in Chinese and European SD cohorts have demonstrated elevated interferon scores compared to healthy controls, indicating activation of type I interferon pathway [13, 34]. Mechanistically, IFNα can induce neutrophil extracellular trap (NET) formation containing oxidized mitochondrial DNA, which in turn amplifies type I interferon signalling, creating a self-perpetuating inflammatory loop [34]. NETs also contribute to NLRP3 inflammasome activation, providing an additional mechanism for the elevated IL-1β and IL-18 levels characteristic of SD [35].

Consistent with these pathways, JAK inhibition with ruxolitinib has shown efficacy in refractory macrophage activation syndrome, likely through suppression of aberrant NETosis and broad inhibition of interferon-driven inflammation [36].

The activation of the type I interferon pathway may reflect viral triggers in disease pathogenesis or genetically determined predisposition for aberrant activation of these pathways.

### Hyperferritinemia

Hyperferritinemia is characteristic of SD, particularly in MAS, and plays an active inflammatory role beyond serving as an acute phase reactant. While earlier studies identified NF-κB activation, recent evidence demonstrates that ferritin binds Msr1 receptors on neutrophils, thereby triggering MAPK-dependent signalling and ROS/PAD4/NE-dependent NET formation that drives cytokine storm (IL-6, TNF-α, MCP-1) and multi-organ inflammation [37, 38]. Blockage of Msr1 or inhibition of NET formation could alleviate disease pathology in experimental models [38].

### mTORC Signalling – A Central Integration Hub

Recent evidence suggests that mTORC1 may act as a key convergence point for multiple inflammatory pathways in SD. Huang et al. demonstrated increased mTORC1 activity in both human SD patients and mouse models, with preferential activation in monocytes. Experimentally, they showed that IL-1β, IL-6, IL-18, and IFN-γ—all cytokines implicated in SD pathogenesis—can each independently induce mTORC1 signalling in myeloid cells, suggesting that mTORC1 serves as a convergence point integrating signals from multiple pro-inflammatory cytokine pathways. This concept was supported by transcriptomic analysis showing that the mTORC1 gene signature in sJIA patients correlated with disease activity and treatment response to canakinumab. Unrestricted mTORC1 activation through genetic deletion of its negative regulator Tsc2 in mice was sufficient to induce SD-like disease with both inflammatory arthritis and spontaneous macrophage activation syndrome with fulminant hemophagocytosis. Conversely, mTOR inhibition with rapamycin prevented MAS development and reduced established inflammation in a mouse model. These findings provide a mechanistic rationale for the use of mTOR inhibitors such as sirolimus in treatment-refractory SD patients [39].

### Adaptive Immune Contributions

While innate immune dysregulation has been the primary focus in SD research, recent single-cell analyses have revealed complex alterations across both innate and adaptive immune compartments. A comprehensive mass cytometry study demonstrated hyperactivation of CD8 + T cells with reduction in CD4 + T cells and B cells in untreated SD patients. Notably, these dysregulated immune profiles were markedly restored following IL-6R blockade therapy, and integration of cytokine profiling revealed strong associations between IL-18, adaptive immune dysregulation, and disease activity [40].

### Autoimmune Phenomena: Neutralizing Anti- IL1Ra Autoantibodies

Neutralizing autoantibodies against the natural antagonist of IL-1, IL1Ra, were found in a proportion (18,75%) of patients with paediatric and adult SD. However, there was no clear association of seropositivity and disease activity or other cytokines like IL-6 or IL-18 and they could not link this finding to non-response to Anakinra. Nevertheless, this is evidence for autoimmune events in a subset of SD patients that might disrupt the fine balance between pro- inflammatory signals and their natural counterparts. However, they could show that anti-IL1 therapies like Anakinra were capable of neutralizing the impairing influence of autoantibodies on IL-1Ra function [41].

### T cell Plasticity and Disease Chronification

There is evidence for an interesting connection between the innate and adaptive immune system in regards to disease progression from acute to a chronic course. This theory evolves around the plasticity of T cells, specifically the instability of FOXP3 expression, the hallmark transcription factor of Tregs. T cells can transdifferentiate from a regulatory phenotype (FOXP3+) into an effector Th17 cell type (FOXP3-) under the influence of cytokines of the innate immune system, specifically IL-6. The groups involved in this research could show that this Th17 polarisation happened during the transition from the acute to the chronic phase of the disease and these Th17 cells are likely playing a role in development of arthritis [42, 43].

The distinction between innate and adaptive immunity is not absolute. Adaptive immune cells can directly engage innate effector mechanisms, as exemplified by CD4 + T cell-induced inflammasome-independent IL-1β production in mononuclear phagocytes, illustrating how inflammatory mediators may be produced through multiple immune compartments [44].

## Clinical Heterogeneity & Recently Recognised Organ-Specific Manifestations

Still’s disease is characterized by four cardinal features: (1) spiking fever (> 39 °C) for at least 7 days, (2) transient salmon-pink rash that often coincides with fever, (3) musculoskeletal involvement such as arthralgia & myalgia or arthritis, and (4) marked systemic inflammation with neutrophilia, raised CRP and ferritin [4]. The disease course is highly variable, ranging from monocyclic patterns with complete resolution to polycyclic relapsing disease and chronic articular courses [45]. However, these manifestations represent the textbook Still’s disease phenotype. In reality, the clinical presentation shows considerable variability, as not all features are present in every patient, and manifestations such as the rash can vary substantially in appearance. This heterogeneity is reflected in existing classification criteria for children & adults (e.g. Yamaguchi & Fautrel, Table 2), which allow for diverse combinations of clinical and laboratory features, resulting in markedly different clinical phenotypes being classified as the same disease entity [46, 47].

**Table 2:** Comparison of the Yamaguchi (1992) and Fautrel (2002) classification criteria for adult-onset still’s disease

Parameter	Yamaguchi Criteria (1992) (46)	Fautrel Criteria (2002) (47)
Total Number of Criteria	8 (4 major + 4 minor)	8 (6 major + 2 minor)
Major Criteria		
1. Fever	≥ 39 °C for > 1 week	Spiking fever ≥ 39 °C
2. Joint involvement	Arthralgias > 2 weeks	Arthralgias
3. Rash	Typical skin rash*	Transient erythema
4. Leukocytosis	WBC ≥ 10 × 10⁹/L with PMN ≥ 80%	PMN ≥ 80%
5. Additonal major	-	Pharyngitis
6. Additional major		Glycosylated ferritin ≤ 20%
Minor Criteria		
1. Throat	Sore throat	-
2. Organomegaly	Lymphadenopathy and/or splenomegaly†	-
3. Liver	Abnormal liver function tests‡	-
4. Serology	Negative RF and ANA¶	-
5. Rash	-	Maculopapular rash
6. Leukocytosis	-	WBC ≥ 10 × 10⁹/L
Exclusion Criteria	**REQUIRED**	**NONE**
	• Infections • Malignancies • Other inflammatory diseases	
Classification Rule	≥ 5 criteria	≥ 4 of the 5 major criteria
	INCLUDING	OR
	≥ 2 major criteria	≥ 3 major + 2 minor criteria

*Typical rash: Macular or maculopapular nonpruritic salmon-pink eruption usually appearing during fever

†Lymphadenopathy: Recent development of significant lymph node swelling, and splenomegaly confirmed on palpation or by echogram

‡Liver dysfunction: Abnormally elevated level of transaminases and/or lactate dehydrogenase, attributed to liver damage associated with this disease but not with drug allergy/toxicity or other causes

RF must be negative by routine test for IgM RF detection, and ANA must be negative by routine immunofluorescence test

All criteria applicable only in absence of other clinical explanations

Recent cluster analyses demonstrate that clinical phenotypes correlate with distinct inflammatory pathways, disease courses, and outcomes, increasingly supporting the hypothesis that Still’s disease encompasses multiple immunological endotypes rather than representing phenotypic variation within a single disease entity [48–50].

Clinical stratification approaches have identified four distinct patient clusters that differ in age of onset, inflammatory markers, organ involvement, and outcomes. Cluster 1 (“Juvenile/Transitional”) comprised predominantly younger patients with high systemic scores and MAS rates but low mortality. Cluster 2 (“Uncomplicated”), the largest group, showed moderate disease activity with the lowest inflammatory markers and represented the typical outpatient phenotype. Cluster 3 (“Hyperferritinemic”) was characterized by extreme ferritin elevation and high CRP but, notably, less multiorgan involvement and predominantly monocyclic disease courses, suggesting that hyperferritinemia alone does not predict poor outcomes. Cluster 4 (“Catastrophic”) comprised older patients with extensive multiorgan manifestations including high rates of lung disease, marked systemic inflammation, and significant mortality [50].

These phenotypic differences likely reflect varying contributions of innate versus adaptive immune mechanisms across different patient subgroups. Organ-specific manifestations further illustrate this heterogeneity.

### Lung Disease

While pleural manifestations have been recognized in Still’s disease for decades, parenchymal lung disease only emerged as a distinct entity in 2013 [51, 52].

The pathogenesis initially appeared linked to IL-1/IL-6R inhibitor therapy, given strong HLA-DRB1*15 associations with drug hypersensitivity reactions during biologic treatment [9, 53]. However, subsequent evidence argues against simple drug causality: 25% of paediatric patients developed lung disease without biologic exposure, and disease severity markers- particularly recurrent MAS, elevated IL-18, and young disease onset- rather than biologic use predicted pulmonary involvement [53, 54].

An alternative cytokine plasticity model suggests IL-1/IL-6 blockade may alter the T cell microenvironment, facilitating shifts in polarization (e.g. TH17◊Th1) that promote IFNγ- driven inflammation independent of hypersensitivity [55].

Mechanistic studies support an IFNγ-dominant inflammatory process. BAL fluid shows elevated IL-18 and interferon-induced chemokines (CXCL9/CXCL10), while lung transcriptomics reveal type II interferon signatures, HLA-D upregulation, and T cell activation—notably present even in histologically normal tissue, suggesting inflammation precedes visible pathology. Histologically, lung biopsies demonstrate lymphocyte-predominant infiltrates (CD4 + > CD8+) with PAP-like features that differ from primary PAP (absent GM-CSF autoantibodies, normal signalling), indicating secondary macrophage dysfunction within an inflammatory milieu rather than primary defects. Variable degrees fibrosis and vasculopathy were also present [54].

Overall, lung disease in Still’s appears driven by adaptive immune mechanisms with substantial overlap to MAS—both show T cell predominance and IFNγ signature, and a history of MAS episodes strongly predicted lung involvement (OR 14.5), suggesting shared pathogenic pathways [54]. The relative contributions of disease-intrinsic inflammation versus drug-modified pathology in HLA-DRB1*15 + individuals remain to be fully defined.

### Cardiac Disease

Recent data from the AIDA Network have characterized cardiac involvement in Still’s disease. While pericarditis is the most common cardiac manifestation, myocarditis occurs in approximately one-quarter of patients with cardiac involvement and is associated with increased mortality, higher systemic scores, and features of severe systemic disease including skin rash and pleuritis [56].

### MAS/HLH – When Autoinflammation Overwhelms

A significant proportion of SD patients develop Macrophage activation syndrome (MAS), a complication which is considered a secondary form of hemophagocytic lymphohistiocytosis (HLH). This hyperinflammatory disease state is associated with significant mortality [57, 58]. Clinically it is characterised by multisystem inflammation with cardinal features being cytopenias, organ dysfunction, coagulopathy and hyperferritinemia. Consistent with its designation as a syndrome, the pathophysiology is likely multifaceted and might be caused by a few different mechanisms.

Generally, MAS is thought to be driven by defective immune regulation, whereby a trigger like a viral infection, in combination with loss of major regulatory feedback loops, for example the termination of immune responses by cytotoxic cell death, leads to an overwhelming immune response by CD8 + T cells and IFNγ, which drives macrophage activation [3]. Notably, these CD8 + T cells are polyclonally rather than clonally expanded, suggesting antigen-independent activation mechanisms that confer innate-like properties and blur traditional boundaries between adaptive and innate immunity [59]. As this vicious cycle remains uninterrupted, sustained activation leads to a cytokine storm and multi-organ failure through systemic inflammation [60].

Studies suggest that a proportion of the patients developing MAS have underlying genetic defects in common primary HLH- associated genes of the perforin- granzyme- pathway of cytotoxicity [61]. This led to the hypothesis of a threshold model of MAS development, in which even mild hypomorphic variants in these genes can lower the threshold so that secondary stimuli, such as infections or inflammatory diseases such as SD can lead to the development of full blown MAS [62]. However, contrary to primary forms of HLH, secondary forms such as MAS in context of SD have shown to not be associated with defective cytotoxicity at least in NK cells [63].

## Therapeutic Implications of the Inflammatory Spectrum

While glucocorticoids remain integral during flares, steroid-sparing strategies have evolved considerably. Randomized trial evidence remains scarce, but available trials and observational cohorts demonstrate superiority of IL-1 and IL-6 receptor blockade over conventional DMARDs and TNF inhibition [4, 64]. Observational data—primarily from pediatric disease—suggest a treatment-responsive “window of opportunity”: early biologics initiation appears to achieve markedly higher remission rates than delayed treatment [65–67]. This observation aligns with the concept of Still’s disease as existing along an autoinflammation-autoimmunity spectrum, wherein early targeting of innate immune dysregulation may prevent progression toward more treatment-resistant chronic disease.

Beyond IL-1 and IL-6 blockade, several emerging therapeutic strategies show promise in treatment-refractory disease. JAK inhibitors have demonstrated efficacy in refractory SD-MAS in Chinese cohorts, likely through broad suppression of multiple cytokine pathways including type I/II interferons [36]. IL-18 binding protein (tadekinig alfa) has shown efficacy in a phase 2 trial, targeting the elevated IL-18 levels characteristic of SD [68]. For severe refractory MAS, IFNγ blockade with emapalumab has shown benefit in an open-label study, mechanistically supported by the central role of IFNγ in MAS pathophysiology [69]. Additionally, the mechanistic evidence of mTORC1 hyperactivation in SD pathogenesis provides rationale for mTOR inhibition, though clinical data in SD remain limited [39].

Although these agents are not yet part of standard treatment recommendations, their mechanistically distinct approaches suggest potential future roles in personalized treatment algorithms.

## Reconceptualizing Still’s Disease: Beyond Classification Criteria

The heterogeneity described throughout this review raises a fundamental question: does Still’s disease represent a discrete entity, or does it function as a clinical repository for systemic autoinflammatory presentations that cannot be attributed to defined diseases?

While the “textbook” Still’s disease patient – presenting with quotidian fever, evanescent salmon-pink rash, and marked systemic inflammation – represents a recognizable phenotype, classification criteria permit considerable diagnostic latitude. The Yamaguchi and Fautrel criteria, for example, designed as exclusion-based frameworks requiring only specific combinations of non-specific features, inevitably capture a broad spectrum of autoinflammatory presentations [46, 47].

This concern is substantiated by clinical observations: patients initially diagnosed with Still’s disease have subsequently been identified as carrying variants in monogenic autoinflammatory disease genes, cluster analyses reveal clinically and prognostically distinct patient subgroups, and marked therapeutic response heterogeneity has been observed across the patient population [11–15, 48–50, 64].

Current classification criteria thus appear to identify a clinical syndrome – characterized by systemic autoinflammation in the absence of alternative explanation- rather than a biologically unified disease. Patients fulfilling the classification criteria may occupy variable positions along the autoinflammation – autoimmunity spectrum (Fig. 1), with presentations reflecting diverse genetic and mechanistic substrates. This nosological ambiguity has practical implications: the diagnostic label “Still’s disease” functions as a pragmatic construct enabling treatment access and clinical management, even as our mechanistic understanding reveals underlying heterogeneity. Future classification approaches incorporating genetic, transcriptomic, or functional biomarkers may enable subdivision into mechanistically defined entities, transforming Still’s disease from a syndrome of exclusion into stratified conditions with targeted therapeutic approaches.

## Conclusion

Still’s disease predominantly reflects autoinflammatory pathology driven by innate immune dysregulation, yet adaptive mechanisms contribute meaningfully to disease heterogeneity and complications. Because it remains a diagnosis of exclusion, careful differentiation from monogenic and other systemic inflammatory disorders is essential. Rather than a single disease entity, Still’s disease may represent a clinical syndrome encompassing multiple pathogenic processes with varying contributions from innate immune activation (inflammasome activation, type I interferon signalling, and acquired clonal hematopoiesis) and adaptive immunity—unified by shared clinical presentations. Recognition as a spectrum disorder with variable innate-adaptive contributions supports unification of pediatric and adult forms and emphasizes the need for biomarker-driven patient stratification to enable mechanism-targeted, personalized treatment approaches.

## Key References

- McGonagle D, McDermott MF. A Proposed Classification of the Immunological Diseases. PLoS Med. 2006 Aug 29;3(8):e297.
  - Conceptual framework for the autoinflammation-autoimmunity continuum.
- Jordan MB, Hildeman D, Kappler J, Marrack P. An animal model of hemophagocytic lymphohistiocytosis (HLH): CD8+ T cells and interferon gamma are essential for the disorder. Blood. 2004 Aug 1;104(3):735–743.
  - Foundational mechanistic study demonstrating the essential role of CD8+ T cells and IFN-γ in HLH/MAS pathogenesis.
- Fautrel B, Touitou I, Girardin S, et al. EULAR/PReS recommendations for the diagnosis and management of Still's disease, comprising systemic juvenile idiopathic arthritis and adult-onset Still's disease. Ann Rheum Dis. 2024 Dec;83(12):1603–1624.
  - First unified evidence-based guidelines for Still's disease, acknowledging diagnostic challenges and calling for improved classification.
- Topping J, Chang L et al. Characterization of Genetic Landscape and Novel Inflammatory Biomarkers in Patients With Adult-Onset Still's Disease. Arthritis Rheumatol. 2025;77(5):583–594.
  - Comprehensive genetic study revealing both germline autoinflammatory variants and somatic mutations, supporting genetic complexity and a polygenic model.
- Ruscitti P, Cantarini L, Nigrovic PA, McGonagle D, Giacomelli R. Recent advances and evolving concepts in Still's disease. Nat Rev Rheumatol. 2024 Feb;20(2):116–132.
  - Comprehensive synthesis of current pathophysiological understanding, clinical heterogeneity, and evolving classification concepts.

## Data Availability

No datasets were generated or analysed during the current study.
